# Barriers and facilitators to adherence to national drug policies on antibiotic prescribing and dispensing in Bangladesh

**DOI:** 10.1186/s40545-021-00342-7

**Published:** 2021-11-16

**Authors:** Fosiul Alam Nizame, Dewan Muhammad Shoaib, Emily K. Rousham, Salma Akter, Mohammad Aminul Islam, Afsana Alamgir Khan, Mahbubur Rahman, Leanne Unicomb

**Affiliations:** 1 Environmental Interventions Unit, Infectious Diseases Division, International Centre for Diarrhoeal Disease Research, Bangladesh (icddr,b), Dhaka, 1212 Bangladesh; 2 Centre for Global Health and Human Development, School of Sport, Exercise and Health Sciences, Loughborough University, Loughborough, UK; 3 Paul G. Allen School for Global Animal Health, Washington State University, Pullman, WA USA; 4 Directorate General of Health Services (DGHS), Dhaka, Bangladesh

**Keywords:** Antimicrobial resistance (AMR), Irrational antibiotic use, Drug policy, Qualified physicians, Quack/village doctor, Low- and middle-income countries (LMICs)

## Abstract

**Background:**

The National Drug Policy in Bangladesh prohibits the sale and distribution of antibiotics without prescription from a registered physician. Compliance with this policy is poor; prescribing antibiotics by unqualified practitioners is common and over-the-counter dispensing widespread. In Bangladesh, unqualified practitioners such as drug shop operators are a major source of healthcare for the poor and disadvantaged. This paper reports on policy awareness among drug shop operators and their customers and identifies current dispensing practices, barriers and facilitators to policy adherence.

**Methods:**

We conducted a qualitative study in rural and urban Bangladesh from June 2019 to August 2020. This included co-design workshops (*n* = 4) and in-depth interviews (*n* = 24) with drug shop operators and customers/household members, key informant interviews (*n* = 12) with key personnel involved in aspects of the antibiotic supply chain including pharmaceutical company representatives, and model drug shop operators; and a group discussion with stakeholders representing key actors in informal market systems namely: representatives from the government, private sector, not-for-profit sector and membership organizations.

**Results:**

Barriers to policy compliance among drug shop operators included limited knowledge of government drug policies, or the government-led Bangladesh Pharmacy Model Initiative (BPMI), a national guideline piloted to regulate drug sales. Drug shop operators had no clear knowledge of different antibiotic generations, how and for what diseases antibiotics work contributing to inappropriate antibiotic dispensing. Nonetheless, drug shop operators wanted the right to prescribe antibiotics based on having completed related training. Drug shop customers cited poor healthcare facilities and inadequate numbers of attending physician as a barrier to obtaining prescriptions and they described difficulties differentiating between qualified and unqualified providers.

**Conclusion:**

Awareness of the National Drug Policy and the BPMI was limited among urban and rural drug shop operators. Poor antibiotic prescribing practice is additionally hampered by a shortage of qualified physicians; cultural and economic barriers to accessing qualified physicians, and poor implementation of regulations. Increasing qualified physician access and increasing training and certification of drug shop operators could improve the alignment of practices with national policy.

## Background

Antimicrobial resistance (AMR) is a great concern for low- and middle-income countries (LMICs) including Bangladesh, often driven by irrational antibiotic prescribing, dispensing and use, for both humans and animals. In common with many other LMICs, there is a lack of compliance with government policy for antibiotic prescription in Bangladesh [1–3]. This is an important factor contributing to the overuse and misuse of antibiotics that are widely available over-the-counter for humans and animals [4, 5]. Additionally, unqualified practitioners, who have no health-related training (e. g. traditional healers, traditional birth attendants, village doctors, drug sellers) are the major (88%) providers for the poor and disadvantaged [6]. Antibiotics provided to patients without prescription are more likely to be used inappropriately, taken for an incorrect duration (course), or at the wrong dose [7]. Studies from Bangladesh have revealed that antibiotic prescriptions were most often issued for children aged 0–15 years (35%) followed by over 60-year olds (23%) [8]. The same study estimated 29% of prescriptions were from qualified doctors (MBBS), whilst 63% came from unqualified practitioners [8]. Informal drug sellers (unqualified private entrepreneurs) also fall victim to aggressive marketing strategies from pharmaceutical companies resulting in overprescribing; multidrug prescribing; using unnecessarily expensive drugs, and dispensing drugs without a prescription [9].

The Government of Bangladesh National Drug Policy, 2016 has as its two primary objectives ‘(t)o ensure people can have easy access to safe, effective and good quality drugs at affordable prices’ and ‘(t)o ensure rational and safe use of drugs and proper dispensing’. The policy outlines the requirements for access to quality drugs and skilled physicians and veterinarians to ensure human and animal health and to line up with national policies on health and population, to replace the policy from 2005. The policy encompasses the ‘safe and rational use of drugs’ and regulatory aspects of the national regulatory body, the Directorate General Drug Administration (DGDA). The only text on antibiotics refers to (a) hospitals developing ‘antibiotic user guidelines’ and (b) the formation of hospital committees to ensure rational use but there is no mention of AMR. According to the National Drug Policy, drug shops can dispense medicines that are registered by the DGDA but antibiotics and other prescription drugs can only be dispensed against a registered physician’s prescription, as a full course [10]. In 2016 a document outlining a pilot for model pharmacies and model drug shops was drafted (‘Standards for the Establishment and Operations of Model Pharmacies and Model Medicine Shops) known as Bangladesh Pharmacy Model Initiative (BPMI) [11]. The pilot planned to include 30 level I pharmacies and 2000 level II pharmacies selected from five divisional towns and six sub-districts for accreditation. This initiative was driven by the National Drug Policy, 2016 in terms of recommended staff qualification, shop registration, standards for premises, dispensing practices, medicine storage, hygiene, record keeping, medicine disposal, allowable products and services, necessary reference materials and drug pricing. The document referred to inspections and that premises and practices would be subjected to penalties under ‘existing acts’. The government-led BPMI has a two-tiered system for ‘model’ retail drug outlets: (1) Model pharmacies, which are managed by a Grade A pharmacist (MPharm or BPharm qualification) on the premises and (2) Model Medicine Shops which have, as a minimum, a person with a C grade pharmacy qualification (12-week training certificate). A B grade pharmacist can work as an assistant to a grade A pharmacist at a Model Pharmacy, and can also work for Model Medicine Shops. [11]. The DGDA has appointed inspectors in each district to visit and supervise drug shops.

This paper reports on the study objective to explore the awareness of relevant policies and guidelines among drug shop operators and customers. Further, it identifies current dispensing practices and the barriers and facilitators to adherence to policies on antibiotic prescribing and dispensing.

## Methods

### Study site and sampling

From June 2019 to August 2020, we conducted a qualitative study in one rural area (Kaliganj sub-district of Gazipur District) and one urban area (Rupganj sub-district of Narayanganj District). The areas were outside the major cities which tend to be over-represented in research. The sites were broadly representative of the urban and rural settings across much of Bangladesh in terms of having a range of drug shop types, size, and serving households of different income levels. Moreover, both sites were within reasonable travel distance from the research institute (in the capital city, Dhaka) which therefore aided field activities and data collection.

After selecting the study areas, we carried out a transect walk of each local bazaar and shopping area. We noted the total number of retail drug outlets of each bazaar and used convenience sampling to select shops from the bazaar with the highest number of drug shops (rural = 25, urban = 32), and invited all drug shop operators to participate in the study. Among the 25 rural and 32 urban drug shops, some refused to participate in the workshop; a total of 35 attended the workshop.

We enrolled households from the villages and urban areas adjacent to the enrolled drug shops that agreed to participate in the study. We purposively selected households with children under 5 years and older family members, groups at increased risk of illness. To select households, we visited each house and approached them to participate in the study. We selected households from two socio-economic strata using a threshold monthly income of below or above Taka 15,000 (USD ~ 192) based on the national mid-range household income (Bangladesh Bureau of Statistics Census, 2016) to ensure socio-economic variability. This also aimed to provide maximum variation in the social context of the sample. Household head/decision-maker and/or primary caregivers were invited to take part in the workshop/interviews.

### Data collection

We conducted this study employing qualitative methods to assess the level of awareness of the National Drug Policy, 2016 among drug shop operators and customers, and barriers and facilitators to adherence.

We organized a stakeholder working group meeting with nine purposively selected members representing key actors in informal market systems namely: government representatives, those from the private sector, not-for-profit sector and membership organizations [12]. Specifically, we included representatives involved in the Bangladesh Pharmacy Model Initiative (BPMI) rollout, including senior staff (*n* = 2) from the DGDA, the regulatory authority members responsible for licensing drug shops, a member of the Bangladesh Chemist and Druggist Association, the pharmaceutical industry (*n* = 2), a consumer association in Bangladesh, a pharmacy owner and a local NGO representative. Subsequently, we conducted four co-design workshops in total, one workshop with household members and one with drug shop operators in each of the rural and urban sites. Co-design is a recognized methodology for developing community-based interventions tailored to the specific context where they will be implemented [13]. We conducted in-depth interviews with 12 household members (as drug shop customers) and 12 drug shop operators; for both respondent groups, six from urban areas and six from rural areas, who were not enrolled in other data collection. We conducted key informant interviews with 12 key personnel involved in aspects of antimicrobial resistance, e.g., from a pharmaceutical company, model drug shop owner/operator and research staff with expertise in antimicrobial resistance and/or intervention development; and a group discussion with stakeholders representing key actors in informal market systems namely: government representatives, private sector, not-for-profit sector and membership organizations.

Interviews and workshops were conducted in Bangla language, where researchers used a guideline that included a range of topics covering our study objectives; for drug shop owners or operators we recorded drug shop license status. Researchers recorded all data using digital audio recorders and also took notes during co-design workshops and group discussions. The data collection was led by a sociologist and the data collection team included anthropologists and social science graduates who were trained and experienced in conducting qualitative research. Before data collection started, as a team we thoroughly reviewed the research objectives, research tools and specific data collection techniques. To ensure uniform understanding among all data collectors, we provided in-house training including mock test exercises, and pre-testing in the field with the data collection instruments for a week.

### Data analysis

The audio recordings were transcribed verbatim in Bangla language. Two anthropologists analyzed the qualitative data using the rules of content and context analysis [14]. Codes and sub-codes were identified according to our research objective and summarized in English by listening to data on the recordings. Each of the interviews and discussions were separately summarized following the same method. We compiled all data under each theme for all the interviews/discussions separately, then the thematic framework was shared with the wider team for discussion and agreement. Following this, thematic content analysis was carried out to provide a brief description of our findings. No attempt was made to quantify study findings, as the number of interviews was small.

**Fig. 1:**
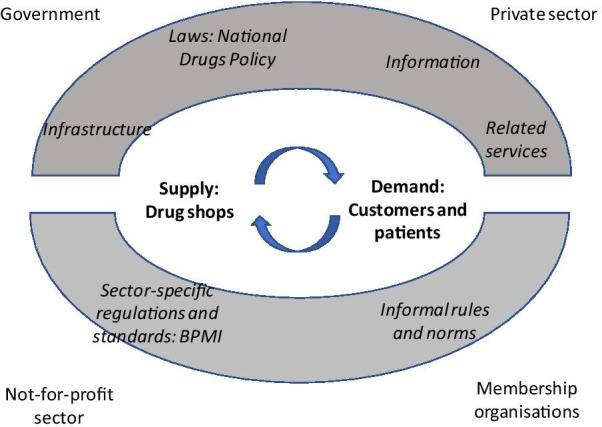
Conceptual framework of market systems pertaining to informal healthcare provision through drug shops (adapted from Bloom et al. [12])

We analyzed data using the conceptual framework of market systems pertaining to informal systems following Bloom et al. [12], which we adapted to the context in Bangladesh (Fig. 1). Drug shops were placed at the center as the market suppliers, and their customers or patients (household members) as the market demand. We considered the antibiotic prescribing specifics of the National Drug Policy as the central policy framework and the Bangladesh Pharmacy Model Initiative as the sector-specific guideline (Fig. 1).

### Ethical considerations

The protocol for this study was reviewed and approved by the icddr,b Institutional Review Board (Protocol # PR-19077) and WHO Ethics Review committee (# 004736). Before taking part in the study, participants were provided with written study information in Bangla which was read aloud to them and they were given an opportunity to ask questions. If they agreed to be interviewed, they were asked to sign a written informed consent form.

## Results

We have presented our study findings using a thematic data analysis approach based on our research objectives; participants characteristics; current antibiotic dispensing practices in relation to National Drug Policy; cost as a barrier for customers; confusion over who can prescribe; barriers to prescribing a full course of antibiotics; lack of awareness of the National Drug Policy; limited formal healthcare facilities for patients and customers. Deductive themes were derived from the conceptual framework and existing literature combined with inductive themes identified from the interview and workshop transcripts. Under each theme, first we state findings from household members then drug shop operators and then from the stakeholders (stakeholder meeting and KII), where applicable.

### Participant characteristics

The study enrolled different participants for each component, i.e., co-design workshops, in-depth interviews, stakeholder meeting and key informant interviews (Table 1). Among the drug shop co-design participants, all were male except for one female; we rarely observed female-run drug shops in the study areas. Regarding the level of training, 14 out of 47 drug shop operators who participated in workshops and in-depth interviews reported having no health-related training. In the in-depth interviews with drug shop operators, 10 out of the 12 participants had completed some form of institutional training such as the Rural Medical Practitioner training (RMP) or Local Medical Assistant and Family Planning (LMAF) courses. The RMP and LMAF courses are of 3–12 months duration. These courses provide basic knowledge on 5–7 subjects, including human anatomy, physiology, pharmacology, practice of medicine, first aid, preliminary microbiology and pathology, and family planning. A secondary school graduate (class 10) can enroll in these courses (https://www.jeeon.co/rural-medical-practitioners-the-first-line-of-defense-in-rural-healthcare-part-2/). Health-care providers with these qualifications are also known as ‘village doctors’ in the community (Table 1). The stakeholder working group meeting consisted of nine members from different organizations including the representatives of government health authorities (Table 1). Among the 12 key informants, eight were social science researchers and public health professionals, and four had a medical degree (Table 1).

**Table 1:** Socio-demographic information of study participants

Categories	Stakeholder (*n* = 9)		Key informant (*n* = 12)	Drug shop					Household/customer			
				Co-design workshop (*n* = 35)			In-depth interview (*n* = 12)		Co-design workshop (*n* = 30)		In-depth interview (*n* = 12)	
				Urban (*n* = 22)		Rural (*n* = 13)	Urban (*n* = 6)	Rural (*n* = 6)	Urban (*n* = 15)	Rural (*n* = 15)	Urban (*n* = 6)	Rural (*n* = 6)
Gender												
Male	7		8	21		13	6	6	15	15	3	3
Female	2		4	1		0	0	0	0	0	3	3
Age												
20–30	–			3		1	3	2	7	2	2	3
31–40	1		6	9		2	2	2	5	1	3	2
41–50	7		5	9		4	1	1	2	1	1	1
51–60	1		1	0		6	0	1	1	4	0	0
More than 61 years	0		0	0		0	0	0	0	7	0	0
Education												
No education	0		0	0		0	0	0	0	1	0	0
Grade 1–10	0		0	10		9	1	2	2	4	4	0
Secondary	0		0	8		4	3	1	5	3	1	2
University/diploma	9		12	4		0	2	3	8	7	1	4
MBBS	0		4	0		0	0	0	0	0	0	0
Organization												
Government		4	0	–		–	–	–	–	–	–	–
Non-Governmental Organization (NGO)		1	3	–		–	–	–	–	–	–	–
Pharmaceuticals company		2	1	–		–	–	–	–	–	–	–
Bangladesh druggist and chemist sanity		1	0	–		–	–	–	–	–	–	–
Drug shop		1	0	–		–	–	–	–	–	–	–
Model pharmacy		0	1	–		–	–	–	–	–	–	–
Consultancy firm (visual media)		0	2	–		–	–	–	–	–	–	–
Research staff		0	5	–		–	–	–	–	–	–	–
Training												
No training		–	–	9		3	2	0	–	–	–	–
Paramedic course (4 years)		–	–	1		4	0	2	–	–	–	–
RMP (rural medical practitioner)/LMAF course		–	–	7		4	4	5	–	–	–	–
Pharmacy course		–	–	5		2	0	0	–	–	–	–
Model pharmacy training		–	–	0		0	0	0	–	–	–	–
Dentist		–	–	1		0	0	0	–	–	–	–
Doctor visit drug shops												
Doctor visits		–	–	4	3		1	0	–	–	–	–
Doctor not visits		–	–	17	0		0	0	–	–	–	–
Drug shop respondent type												
Drug shop owners (also operate)		–	–	20	12		3	6	–	–	–	–
Drug shop operators		–	–	1	1		3	0	–	–	–	–
Experience (years)												
0–10		–	–	–	–		5	4	–	–	–	–
11–20		–	–	–	–		1	1	–	–	–	–
21–30		–	–	–	–		0	1	–	–	–	–
Having license												
License		-	-	15	6		5	2	–	–	–	–
No license		-	-	7	7		1	4	–	–	–	–
Household income (monthly) < 15,945 Taka (USD ~ 192)												
Decision-maker/caregiver of the household		–	–	–	–		–	–	6	5	3	3
Households with child < 5 years old		–	–	–	–		–	–	2	2	2	3
Households with older family member > 65 years old		–	–	–	–		–	–	3	4	4	3
Household income (monthly) > 15,945 Taka (USD ~ 192)												
Decision-maker of the household		–	–	–	–		–	–	9	10	3	3
Households with child < 5 years old		–	–	–	–		–	–	6	6	1	1
Households with older family member > 65 years old		–	–	–	–		–	–	6	5	2	1

### Current antibiotic dispensing practices in relation to National Drug Policy

The National Drug Policy, 2016, is specific on the need for a prescription from a qualified physician. Drug shop co-design participants reported that drug shop operators frequently dispense and recommend antibiotics despite the fact that they were aware of the government rule that only registered doctors can perform this function. A government registered physician (who also managed a drug shop) described this common practice among drug sellers and questioned their ability to prescribe antibiotics. He argued that antibiotics are available in all drug shops but not all the drug sellers know which antibiotic will be effective for patients or illnesses.

*“Drug shop operators dispense antibiotics based on the assumption that patients will get better without knowing whether it is needed or not”*

*(Drug shop operator, co-design workshop participant, rural site)*

Nonetheless, drug shop operators held varied views on what constituted appropriate antibiotic use. During co-design workshops, one participant from the urban site stated that in many cases, different drug sellers are providing different courses of antibiotics to the patients.

*“We are (drug shop operators) selling and prescribing medicines to the patients. When the patients do not recover, they come to us again for further treatment. In some cases, some of us change the medicine including antibiotics several times. This kind of practice of changing drugs creates resistance in the body. I think this is one of the main reasons of drug resistance. This may not seem important to many of us now. However, this might cause great damage for our future generation regarding getting cured from diseases.”*

*(Drug shop operator, co-design workshop participant, urban site)*

Interviews and workshop discussions also identified that the drug shop operators had no clear knowledge of different antibiotic generations, how and for what diseases antibiotics work contributing to inappropriate antibiotic dispensing. Drug shop operators reported that they know there are different generations of antibiotics, but lacked adequate knowledge about the specific names of the antibiotics according to their generations, and did not know how to select the appropriate generation of antibiotics for patients.

*“We mostly prescribed third generation (beta-lactam) antibiotics. But we do not know which generation of medicine to provide to different patients. Moreover, we do not know why we need to prescribe this generation of antibiotics. We do not have any in-depth knowledge neither on antibiotics generation nor how to prescribe antibiotics based on generation. We need training on this.”*

*(Drug shop operator, co-design workshop participant, urban site)*

During key informant interviews, a pharmaceutical company representative reported that their company usually informs registered doctors about the latest generation of antibiotics and related prescribing guidelines, but it is not part of their remit to inform drug shop operators about the prescribing guidelines, only registered doctors. Although not reported in our interviews and workshops, other studies have reported that pharmaceutical representatives are an important source of knowledge on new antibiotics and prescribing for drug sellers [12, 15].

*“We always keep doctors up-to-date about the new generation's antibiotic we produce. We are doing everything we can. Doctors are aware of policies. As our promotions do not include drug shop operators, we can’t do much for them”*

*(Stakeholder, key informant interview participant)*

During the meeting with stakeholders, government personnel reinforced that there is no alternative to the National Drug Policy which prohibits the sale and distribution of antibiotics without prescription from a registered physician, and that drug shop operators have to maintain this to avoid regulatory action/punishment.

### Cost as a barrier for customers

A common reason reported by household participants for visiting a drug shop first to discuss illness and therapy, rather than qualified doctors, was to avoid the cost of a qualified physician’s consultation that often included further inconvenience and costs for various diagnostic tests, whereas consultation fees were not associated with visiting drug shops.

*“For fever, cough, and dysentery-I go to a local drug shop for seeking healthcare. The treatment cost is low in drug shop and it is nearby. Hospitals are far away from my area. If we get better from drug shop's treatment, then we do not visit any doctor”*

*(Household member, in-depth interview participant, urban site)*

*“I think that the pharmacists in our area are providing better services compared to a specialist doctor (qualified doctor). Because they do not give people a hard time with various diagnostic tests”*

*(Household member, co-design workshop participant, urban site)*

Customers reported that cost was also why they did not buy the full course of antibiotics, which was similarly reported by drug shop operators. In the stakeholder group meeting and in the key informant interviews, cost as a barrier to purchasing a full course was also raised; stakeholders stated that there is a need to motivate economically disadvantaged people to buy the full course of antibiotics.

*“From my working experiences, I have seen many drug sellers tell people to purchase the full antibiotic course. But patients think it is a waste of money as they get better after taking 2 or 3 antibiotic tablets. Financial circumstances are the main constraint which forces people to shop for a single antibiotic tablet. However, most of the people know that completing the full course antibiotic is mandatory for better health".*

*(Stakeholder, key informant interview participant)*

### Confusion over who can prescribe

Customers had difficulties differentiating between qualified and unqualified physicians. Also, customers had varied understanding about who is qualified to prescribe antibiotics. The rural household co-design workshop participants recognized MBBS doctors (Bachelor of Medicine-Bachelor of Surgery) or higher medical degree holders as “qualified doctors”. However, regarding the RMPs or ‘village doctors’, participants had doubts about whether they are qualified or not. While some of the participants were doubtful about the medical knowledge of RMPs to prescribe antibiotics, others argued that the RMPs are also registered, and perceived them as qualified doctors, and thus have the authority to prescribe antibiotics.

*“What about the rural medical practitioners? As they are also registered. Patients take antibiotics by following their (RMPs) prescriptions”*

*(Household member, co-design workshop participant, rural site)*

Based on our observations, we understood that many of the drug shop operators considered themselves as equally or better qualified than the Sub-Assistant Community Medical Officers (SACMOs) and/or community healthcare providers (CHCPs). In fact, some of the participating drug shop operators had completed training as SACMOs or CHCPs. SACMOs and CHCPs are government employees and authorized to prescribe a limited number of antibiotics. SACMO have 3 years of training as a diploma course on clinical diagnosis and prescription, and CHCPs have 3 months of basic training on management of common health conditions. Respondents argued that if a SACMO or CHCP can advise on antibiotics with their limited training, why can’t they? Thus, they continue to prescribe.

### Barriers to prescribing a full course of antibiotics

In terms of completing the full course of antibiotics, household participants indicated that they needed the opportunity to understand the importance of completing the full course of antibiotics and following the instructions for the prescription.

The drug shop operators reported that many of them asked their patients to take the full course of antibiotics as they consider it necessary to sell the full course, which is good for their business in terms of both reputation and profit. However, by recommending the full course, they sometimes faced the challenge of losing customers. Although sellers encouraged customers to buy the full course most refused because they considered that the full course is not needed or the drug shop operator is prescribing it unnecessarily.

Buyers and consumers have a wide choice of antibiotic providers from the many drug shop outlets which makes it simple to find an alternative shop that will provide the service they need. Hence, drug shop operators were concerned that if they advise against taking antibiotics, or insist on dispensing only with a prescription, the customer may take their business to another shop where they can easily buy antibiotics.

*“If I do not want to sell antibiotics (without registered doctor’s prescription) to anyone, they think I am scared about drug reaction or I have no knowledge about the drug. Consumers do not hear us at all. If I recommend them not to take antibiotics, they go to another drug shop and buy their desirable drug. This is a common scenario. So, building awareness among consumers is important”*

*(Drug shop operator, in-depth interview participant, rural site)*

Regarding incomplete course taking, the participants reported that one of the main reasons that patients did not comply was that their symptoms resolved after a few days of antibiotic consumption.

*“Many people do not finish the full course of antibiotics. After following the medication for 2 or 3 days, if people feel better and healed, they stop taking the remedies. People do not realize how important it is to complete an antibiotic course.”*

*(Household member, co-design workshop participant, urban site)*

### Lack of awareness of the National Drug Policy

Most of the drug shop operators had not heard of the Bangladesh Pharmacy Model Initiative (BPMI), which includes guidelines on staff qualifications premises and location, dispensing practices, storage, hygiene, documentation system, medicine disposal and other related guidelines.

The stakeholder working group participants stated that the Directorate General of Drug Administration (DGDA) had been actively motivating drug shops to be part of the BPMI program, but it is an ongoing challenge to involve all drug shops under this initiative. Some key informants also described knowledge gaps among drug shop operators, such as poor awareness of the BPMI, and indicated a lack of impact of the BPMI on drug shop operator practices. However, at the time the study was conducted only 347 model drug shops had been included in the program. One key informant thought that although the government has a monitoring system to detect whether drug shops have licenses and are being operated according to the guideline, the system is not functioning properly due to a shortage of resources.

*“DGDA is centrally monitoring whether legislation is being implemented or not. DGDA has appointed inspectors in each district to supervise the situation. DGDA keeps a check on drug shop operators if they are maintaining proper temperature of drug and selling licensed as well as expired drugs. Due to limited manpower, DGDA claims they cannot operate frequently and effectively”*

*(Stakeholder, key informant interview participant)*

Nonetheless, most of the drug shop operators in this study had some form of institutional training. Based on their training through various short certificate courses as rural medical practitioner (RMP) or similar (6–12 months of training on medicine dispensing), they considered that they should have the right to prescribe antibiotics. Participants voiced concerns about government legislation whereby rural medical practitioners (village doctors) are not allowed to prescribe antibiotics and argued that the government must remove this bar as drug shops play a pivotal role in the health care system. One respondent described his concerns about the government introducing laws that do not consider the social context of sellers currently prescribing antibiotics:

*“If the government introduces laws without thinking about rural medical practitioners/village doctors, it will not be appropriate.* We *want to learn the proper use of antibiotics and we will carry our learning to the people, it is our responsibility. Government’s responsibility is to train us through various conferences, seminars, and training (on the appropriate use of antibiotics)…..”*

*(Drug shop operator, co-design workshop participant, urban site)*

### Limited formal healthcare facilities for patients and customers

Residents in the urban and rural areas had a range of health care facility options including private hospitals, Union Health and Family Welfare Centres (UHFWC), drug shops, and government health complexes. However, the participants raised concerns about the standard of the available healthcare facilities and the numbers of qualified physicians available at those facilities which they felt were inadequate. Also, they highlighted that medicines were often unavailable from government facilities.

*“There is a scarcity of qualified doctors (in our locality) and there are no standard government health facilities like medical college hospitals. Also, the unavailability of medicine is a common issue in the government health facility center”*

*(Household member, co-design workshop participant, urban site)*

Access to qualified physicians was a significant challenge due to the scarcity of fully trained doctors. Participants reported that for minor illnesses like cold, fever, cough, gastrointestinal problems, and small cuts they visit drug shops to get treatment, advice and medicine. For these illnesses, drug shop operators usually provided medicine for 3–5 days and advised patients to come back later for follow-up treatment advice.

*“People usually come with normal diseases like fever, flu, upset stomach (diarrhea), ulcer, etc. Children come with diarrhea usually. Adults come with a gastric problem as well as complaining about weaknesses that they do not have the physical strength to do any work”*

*(Drug shop operator, co-design workshop participant, rural site)*

## Discussion

There are social norms involved in customer health seeking behaviors such as the expectation of free consultation opportunities from drug shops, and the convenience of visiting local unqualified drug shop staff in contrast to qualified doctors; this can contribute to poor antibiotic stewardship, which is similarly found in other LMICs [12, 16]. An over-supply of drug shops in the informal market means that competition for customers is high. This is a barrier for drug shop operators to follow prescribing guidelines or comply with antibiotic stewardship for fear of losing their customer base [12, 15, 17–19]. By informing doctors about the latest generation of antibiotics and related prescribing guidelines, the pharmaceutical companies understand that such knowledge will eventually increase demand among patients resulting in inappropriate dispensing practices. There is a lack of understanding among customers about who is a ‘qualified physician’, and therefore permitted to prescribe antibiotics, compared to those who are rural medical practitioner/village doctors. This is an additional determinant of poor compliance with the National Drug Policy.

In order to improve the practices of the drug shop operators and pharmacists, DGDA introduced the Bangladesh Pharmacy Model Initiatives (BPMI) which has so far accredited a small proportion of the estimated 200,000 shops. The ‘model’ is aimed to ensure that drug shops maintain high standards of practice [10, 20]. However, previous surveys among model pharmacies show that they are not necessarily following all the BPMI guidelines [21, 22]. Furthermore, stricter government regulation and enforcement does not always bring about the desired improvements in service provision within the informal sector. It requires actions from all the four components in the informal healthcare market system (government, private sector, not-for-profit sector and membership organization) to strengthen and improve the important role of drug shops. In our study, multiple stakeholders reported limited implementation of BPMI, among the approximate 200,000 drug shops and inadequate monitoring, both of those participating in the BPMI and those who were not. None of the drug shops participating in this study was part of the BPMI program, thus drug shop operators (with one exception) had not received training on BPMI. Additionally, almost no drug shop operators had heard the term ‘model pharmacy’ which indicates a limited penetration of the BPMI initiative. Wider dissemination of the BPMI needs more serious consideration. A rigorous study in the future to assess BPMI implementation would provide important insights and identify ways to improve and disseminate the program on a larger scale. Also, extensive advocacy activities need to be introduced, in collaboration with key stakeholders, to direct and fund BPMI implementation, or to seek alternatives to actively involve drug shops in the process to ease the burden of the formal health sector.

The shortage of qualified physicians per population in Bangladesh with a ratio of 0.6 doctors per 1000 people, as opposed to the standard ratio of 1:1000, with gross imbalance in distribution favoring urban areas has been previously identified [23–25]. Thus, drug shop operators contribute substantially in responding to the health needs of the population and serve as an essential means to cover some of the gaps in the formal healthcare delivery system in Bangladesh [26]. Drug shops play a similar role in other LMICs [27–29]. Monitoring sales of antibiotics without a prescription from a registered healthcare professional will continue the burden on the already overwhelmed government DGDA. The current government policy allows some trained community healthcare professionals, such as -SACMOs and CHCP to prescribe a limited number of antibiotics (Appendix 1) as a part of primary care.

A social and behavioral change communication intervention to encourage customers and drug shop operators to prescribe and consume antibiotics responsibly can provide a bridge to more responsible antibiotic stewardship while health systems are strengthened. Further formative research could provide additional input towards developing a culturally appropriate intervention. As an example, a study conducted in Uganda found that the AXEX (access and excess) intervention improved pediatric fever management using health technologies and the perceived efficacy of medicines was successful. It additionally improved regulator- drug seller interaction, and care-seeking behavior [17].

Though drug shop operators considered themselves more experienced than the SACMOs/CHCPs to prescribe antibiotics, there are counter-arguments against drug shop operators selling antibiotics without prescription from a qualified doctor. We identified considerable shortfalls in their knowledge on antibiotic generations, course, dose and appropriate use in accord with other study findings [6, 7]. A study of medicine dispensing from drug shops in southern Bangladesh (all medicines, not antibiotics specifically) reported that 18.4% of drugs used for common illnesses were appropriate, 7.1% were harmful, and 74.5% were unnecessary but not harmful [12].

The vast gap between the National Drug Policy requirements for antibiotic dispensing with a doctor’s prescription and the common practice of dispensing by providers with minimal qualifications cannot be remedied overnight. It will take considerably more than increasing policy knowledge among drug shop operators and the public on prescription requirements to close the gap. While the government’s policy objectives to allow equitable access or medicines and ensuring rational and safe use are laudable, they are clearly not practical in the short term. Interim steps that move practices in the direction of dispensing antibiotics only when a prescription is presented are urgently needed. It is now 5 years since the policy was formulated and a revision could include a section devoted to antibiotics and antibiotic resistance and that balances access and stewardship. The government could consider a model such as the accredited drug dispensing outlet (ADDO) program, developed in Tanzania by collaboration among the health department and stakeholders, including those involved in regulation [29]. The public–private accreditation program components include changing staff dispensing behavior through training and supervision that allows them to sell essential medicines, including selected prescription drugs. This model has proven successful in several African countries and it is worth considering adaptation to the context in Bangladesh.

## Limitations

The study had a limited number of participants and only included one urban and one rural site. Nonetheless, we recruited national stakeholders and other community participants from diversified socio-economic backgrounds to gain perspectives from a range of sources. We selected sites likely to be representative of urban and rural settings in Bangladesh, however, it is possible that our participants did not represent all communities across the country. We had refusals for participation in the co-design workshops. It is possible that those who refused were systematically different from participants and thus responses could be biased.

## Conclusion

Awareness of both the National Drug Policy and the BPMI was limited among urban and rural drug shop operators; increased knowledge and enforcement could enhance antibiotic stewardship. We identified several barriers to adherence to policies. Moreover, by developing more realistic National Drug Policy could also help strengthen stewardship. To develop an effective National Drug Policy, the World Health Organization recommends involving all related interested groups and stakeholders including other ministries (higher education, trade, industry), doctors, pharmacists and nurses, local and international pharmaceutical industries, drug sellers, academia, nongovernmental organizations (NGOs), professional associations and consumer groups. WHO also recommends that the National Drug Policy committee meets regularly to review the implementation of the policy with all interested parties in a National Drug Policy forum [30]. Poor stewardship is additionally hampered by a shortage of qualified physicians; cultural and economic barriers to accessing qualified physicians and insufficient national implementation of the BPMI. Therefore, to prevent drug shop operators from dispensing antibiotics without prescription in the near future would be challenging without increasing the availability of qualified physicians and/or trained SACMO/CHCP, and strengthening knowledge using a social and behavioral change communication program. Political commitment is needed to prioritize developing a realistic drug policy that can facilitate responsible antibiotic stewardship. Further broadening the BPMI implementation should be given a high priority.

## Data Availability

The datasets used and/or analyzed during the current study are available from the corresponding author on reasonable request.

## Appendix 1

See Table

**Table 2:** List of antibiotics that SACMO and CHCP are permitted to prescribe

	Name of antibiotics	
	Community healthcare providers (CHCP)	Sub-Assistant Community Medical Officers (SACMO)
1	Albendazole	Albendazole
2	–	Amoxiclav
3	Amoxycillin	Amoxycillin
4	–	Benzathine penicillin
5	–	cephalexin
6	–	Ciprofloxacin
7	Doxycycline	Doxycycline
8	–	Erythromycin
9	–	Gentamycin
10	Metronidazole	Metronidazole
11	Neomycin sulphate with bacitracin	Neomycin sulphate with bacitracin
12	Phenoxymethyl penicillin	Phenoxymethyl penicillin
13	Primaquine	Primaquine
14	–	Procaine Penicillin
15	Rifampicin^a^	Rifampicin
16	Rifampicin + isoniazid + pyrazinamide^a^	Rifampicin + isoniazid + pyrazinamide
17	Rifampicin + isoniazid + ethambutol^a^	Rifampicin + isoniazid + ethambutol
18	–	Silver sulfadiazine

^a^CHCPs cannot *initiate treatment but provide follow-up medicine*

2.
